# Serum Concentration of Thyroid Hormones Long-Term after Sulfur Mustard Exposure

**Published:** 2019-05

**Authors:** Sakine MOAIEDMOHSENI, Tooba GHAZANFARI, Ensie Sadat MIRSHARIF, Nayere ASKARI, Zuhair MOHAMMAD HASSAN, Mohammad Mehdi NAGHIZADEH, Soghrat FAGHIHZADEH, Fereidoun AZIZI

**Affiliations:** 1. Immunoregulation Research Center, Shahed University, Tehran, Iran; 2. Department of Biology, Faculty of Basic Sciences, Shahid Bahonar University, Kerman, Iran; 3. Department of Immunology, Tarbiat Moddares University, Tehran, Iran; 4. Department of Biostatistics and Social Medicine, Zanjan University of Medical Sciences, Zanjan, Iran; 5. Endocrine Research Center, Shahid Beheshti University of Medical Sciences, Tehran, Iran

**Keywords:** Sulfur mustard, Serum, Thyroid hormones

## Abstract

**Background:**

Despite several reports on the clinical manifestations of sulfur mustard (SM) intoxication, there is no study on serum concentrations of thyroid hormones long-term after SM exposure. In this study, the changes in thyroid functioning parameters 20 yr after SM exposure were evaluated.

**Methods:**

This study is a part of a larger historical cohort study conducted in 2007 following 20 years of the exposure to SM, called Sardasht–Iran cohort study (SICS). We (SICS) comprised an SM–exposed group from Sardasht City, West Azerbaijan Province, Iran (n=169 as hospitalized group and n=203 as non-hospitalized exposed group); and control participants were selected from Rabat, a town near Sardasht (n=126). Peripheral blood samples were taken in fasting state and then the sera were separated. T4, T3, TSH, antithyroglobulin (anti–Tg), and antithyroid peroxidase (anti–TPO) concentrations in the sera were measured by the ELISA method.

**Results:**

The mean of T3 concentration was significantly higher in the exposed than control group (0.88 ± 0.26 nmol/L vs 0.8 ± 0.25 nmol/L, *P*<0.001). The levels of TSH, T4, and T3up were not significantly different between the exposed and control groups. Thyroglobulin level was significantly higher in the exposed non-hospitalized group (56.07 ± 140.22 μg/L vs 17.66 ± 41.49 μg/L, *P*=0.004), but the level of anti–Tg and anti–TPO showed no significant differences between the two groups.

**Conclusion:**

More studies are needed on the alterations in thyroid hormones, their gene expressions, and mechanisms involved in SM exposure to clarify the causes of these alterations.

## Introduction

Sulfur mustard [bis(2–chloroethyl) sulfide; SM], also known as mustard gas, was initially used as a vesicant chemical warfare agent during World War I. According to the report of the specialists appointed by the Secretary-General of the United Nations, mustard gas was extensively used by Iraq during the conflict against Iran (1).

Sardasht, a border town in the northwest of Iran, was exposed to sulfur mustard in 1987 by the Iraqi army. In addition to acute extensive multi-organ problems, there are long-term adverse health effects of exposure to mustard gas (2–4).

Despite a fair number of reports on clinical manifestations of SM intoxication, there are few studies on paraclinical and molecular parameters and systemic effects of SM long term after exposure. Therefore, the underlying molecular mechanisms are less known.

Various complaints and clinical presentations of the survived people seeking medical care and review of the literature support such concerns, even though no systematic scientific evidence has been available (5).

A decline in mean serum–free T4 index (FT4I) and free T3 index was reported less than one month after exposure (6). Moreover, they explained the time course of serum concentration of thyroid hormones. Free T4 and T3 indices decreased and reverse T3 (rT3) increased in the first week following exposure. Except for an increase in FT4I and a decrease in thyroid–stimulating hormone (TSH) by the third week, serum hormone concentrations remained unchanged until the fifth week after injury. They concluded that exposure to chemical weapons containing SM results in alterations in serum concentrations of thyroid hormones.

There is no study on serum concentrations of thyroid hormones long-term after SM exposure. In this study, the changes of thyroid function parameters 20 years after SM exposure were evaluated.

## Materials and Methods

### Study Design and Participants

This study is a part of a larger historical cohort study conducted in 2007 following 20 years of the exposure to SM, called Sardasht, West Azerbaijan Province, Iran cohort study (SICS), of which the details of the study design and methods have been previously reported (7).

Briefly, the exposed group were male individuals from Sardasht categorized into two major subgroups based on severity of injuries, just after exposure. SM–exposed patients hospitalized based on the severity of injuries after acute exposure were considered as the hospitalized group (n=169) who had a history of at least one–week admission in hospital, and patients with mild and subclinical problems not hospitalized after acute exposure as the non-hospitalized exposed group (n=203).

Control participants were selected from Rabat, a town near Sardasht, concurrently with the exposed group. Moreover, the control samples included men matched with the study group by age (n=126). The age range covered by the study was 20–60 yr. Both study groups were compared in terms of marital status, level of education, employment, and smoking status.

### Ethical approval

The study was approved by the Ethical Committee of the Board of Research Ethics of Janbazan Medical and Engineering Research Center (JMERC), the Board of Research of the Ministry of Health and Medical Education, and Shahed University. Volunteers who signed an informed consent were recruited.

### Serum Preparation

Peripheral blood samples were taken in the morning in fasting state using Vacationer tubes (BD Biosciences), 20 years following SM exposure in the exposed group and, alongside, in the control group. After clotting, the sera were separated by a 20 min centrifugation at 2000×g in 4 °C, aliquoted and stored at −80 °C until test.

### Measurement of Thyroid Hormones

T3, T4, and TSH were measured by enzyme-linked immunosorbent assay (ELISA) method (DBC, Canada). Intra- and inter-assay coefficients of variations (CV) were 3.3% and 6.2% for T4, 12.3% and 10.4% for T3, and 3.9% and 7.1% for TSH, respectively.

According to the manufacturer’s instructions, the normal range was 0.52–1.85 ng/ml for T3, 4.4–10.8 μg% for T4 and 0.4–6.1μIU/ml for TSH.

Antithyroglobulin (anti–Tg) and anti-thyroid peroxidase (anti–TPO) concentrations were measured by ELISA (Radim, Pomezia, Italy).

The values of anti–TPO, up to 150IU/ml and anti–Tg, up to 75IU/ml were considered normal. The inter- and intra-assay CVs were 7.2% and 6.5%, respectively.

### Statistical Analysis

Statistical analysis was done using the SPSS (Windows, ver. 16.0, Chicago, IL, USA). Values are given as mean (± SD). Comparison of thyroid factors between study groups was done with ANOVA (Tukey’s post hoc). Comparison of the normal range was done with chi-square test. A *P*–value of less than 0.05 was considered statistically significant.

## Results

The mean age of the exposed group was 44 ± 11 yr. The result revealed insignificant differences in terms of age, marital status, and smoking status in contrast to significant differences in terms of level of education and, and employment, which were higher among the exposed group (Table 1).

**Table 1:** Demographic Characteristics of Study Groups

		***Study Groups***				***P-valve***	***Clinical severity at exposure time***			
		***Control***		***Exposed***			***Non Hospitalized***		***Hospitalized***	
		***N***	***%***	***N***	***%***		***N***	***%***	***N***	***%***
Age(yr)	<40	57	46.0	126	35.3		72	36.9	54	33.3
	40 - 50	35	28.2	113	31.7	0.097	59	30.3	54	33.3
	>50	32	25.8	118	33.1		64	32.8	54	33.3
Marital status	Not-married	9	7.6	28	8.0	0.866	16	8.4	12	7.6
	Married	110	92.4	320	92.0		174	91.6	146	92.4
Education	Lower diploma	93	78.2	201	57.9	<0.001	98	51.9	103	65.2
	Diploma and up	26	21.8	146	42.1		91	48.1	55	34.8
Occupation	Employed	52	44.1	210	60.5	0.002	130	68.8	80	50.6
	Unemployed	66	55.9	137	39.5		59	31.2	78	49.4
Smoke	Yes	29	22.8	84	22.6	0.573	48	23.8	36	21.3
	No	98	77.2	287	77.4		154	76.2	133	78.7

The details of hormonal assessment are presented in Tables 2 and 3. Serum T4 (μg/dL) was 7.95 ± 1.44 (range: 4.4–10.8 μg%) and serum TSH was 1.41 ± 2.06 (range: 0.4–6.1 μIU/ml) in the exposed group. T4 was 8.24 ± 1.78 (range: 4.4–10.8 μg%) and TSH was 1.47 ± 2.19 (range: 0.4–6.1μIU/ml) in the control group.

**Table 2:** Thyroid hormones levels in Sulfur mustard Exposed and control groups

***Study Groups***		***N***	***Mean***	***SD***	***Median***	***P-value^1^***	***P-value^2^***
T3 ng/ml	Unexposed	127	.80	.25	.82		
	Exposed Non Hospitalized	202	.88	.27	.84	0.018	
	Exposed Hospitalized	169	.87	.24	.86	0.058	0.924
	Exposed	371	.88	.26	.85	0.005	
T4 micg/dl	Unexposed	127	8.24	1.78	8.10		
	Exposed Non Hospitalized	202	8.02	1.45	8.10	0.395	
	Exposed Hospitalized	169	7.88	1.43	8.00	0.109	0.665
	Exposed	371	7.95	1.44	8.00	0.068	
TSH mIU/L	Unexposed	127	1.47	2.19	1.01		
	Exposed Non Hospitalized	202	1.61	2.66	1.10	0.809	
	Exposed Hospitalized	169	1.18	.90	.91	0.460	0.110
	Exposed	371	1.41	2.06	1.02	0.805	
T3uptake %	Unexposed	127	28.50	2.27	29.00		
	Exposed Non Hospitalized	202	28.93	2.28	29.00	0.217	
	Exposed Hospitalized	169	28.74	2.26	29.00	0.632	0.712
	Exposed	371	28.84	2.27	29.00	0.140	
Tg ng/ml	Unexposed	121	17.66	41.49	9.40		
	Exposed Non Hospitalized	200	56.07	140.22	11.55	0.004	
	Exposed Hospitalized	166	31.95	82.22	10.50	0.484	0.071
	Exposed	366	45.13	117.98	11.00	0.012	
anti Tg IU/ml	Unexposed	93	81.64	291.88	21.99		
	Exposed Non Hospitalized	134	62.98	141.93	25.32	0.742	
	Exposed Hospitalized	98	43.80	91.95	22.05	0.346	0.722
	Exposed	232	54.88	123.44	24.48	0.001	
anti TPO IU/ml	Unexposed	94	36.56	108.20	7.72		
	Exposed Non Hospitalized	132	29.22	82.34	6.79	0.815	
	Exposed Hospitalized	99	26.16	77.39	7.35	0.697	0.964
	Exposed	231	27.91	80.10	7.20	0.246	
Free T4 Index	Unexposed	127	235.07	53.71	231.40		
	Exposed Non Hospitalized	202	231.34	43.46	230.00	0.763	
	Exposed Hospitalized	169	226.56	45.66	226.20	0.273	0.593
	Exposed	371	229.16	44.48	229.50	0.222	
Free T3 Index	Unexposed	127	22.90	7.52	22.80		
	Exposed Non Hospitalized	202	25.60	8.44	24.38	0.007	
	Exposed Hospitalized	169	25.07	7.22	24.32	0.048	0.791
	Exposed	371	25.36	7.90	24.32	0.002	

*P*-value^1^: Comparison with Control (ANOVA and t-test) //

*P*-value^2^: Comparison between hospitalize and non-hospitalized (ANOVA Tukey Post hoc) //

TSH= thyroid stimulating hormone; Tg=Thyroglobulin; TPO= Thyroid peroxidase

**Table 3:** Comparison of thyroid hormone levels between study groups according to the normal levels

***Variable***		***Unexposed***		***Non-Hospitalized***			***Hospitalized***			***Exposed***		
		***N***	***%***	***N***	***%***	***P-value***	***N***	***%***	***P-value***	***N***	***%***	***P-value***
T3 level (0.52 - 1.85)	< 0.52	15	11.8	10	5.0		6	3.6		16	4.3	
	Normal	111	87.4	189	93.6	0.065	162	95.9	0.023	351	94.6	0.010
	> 1.85	1	.8	3	1.5		1	.6		4	1.1	
T4 Level (4.4 - 10.8)	< 4.4	1	.8	4	2.0		1	.6		5	1.3	
	Normal	121	95.3	193	95.5	0.528	165	97.6	0.513	358	96.5	0.494
	> 10.8	5	3.9	5	2.5		3	1.8		8	2.2	
TSH Level (0.4 - 6.1)	< 0.4	21	16.5	22	10.9		30	17.8		52	14.0	
	Normal	104	81.9	175	86.6	0.300	139	82.2	0.256	314	84.6%	0.767
	> 6.1	2	1.6	5	2.5		0	.0		5	1.3	
T3up Level (25 - 38)	< 25	7	5.5	6	3.0		7	4.1		13	3.5	
	Normal	120	94.5	196	97.0	0.249	162	95.9	0.583	358	96.5	0.320
	> 38	0	.0%	0	.0		0	.0		0	.0	
anti Tg 50–75	lower 75	82	88.2	112	83.6	0.335	91	92.9	0.268	203	87.5	0.868
	75 and up	11	11.8	22	16.4		7	7.1		29	12.5	
anti TPO 100–150	lower 150	89	94.7	126	95.5	0.790	95	96.0	0.674	221	95.7	0.700
	150 and up	5	5.3	6	4.5		4	4.0		10	4.3	

*P*-value: Comparison with Control (Chi-square test)

TSH= thyroid stimulating hormone; Tg=Thyroglobulin; TPO= Thyroid peroxidase

The mean of T3 concentration was significantly higher in the exposed group (0.88±0.26 nmol/L) compared to the control (0.80 ± 0.25) (*P*<0.001); however, these values were still within normal clinical range. The levels of TSH, T4, T3 up, anti–Tg and anti–TPO were not significantly different between the exposed and control groups. Regarding two subgroups of exposure (hospitalized and non-hospitalized), the patterns of measured parameters were similar. Thyroglobulin level was higher in the exposed but not hospitalized group (56.07±140.22 μg/L, *P*=0.004), compared to the control group. The free T3 index, calculated from the serum total of T3 and T3 uptake, was significantly elevated in the exposed group similar to the T3 level. The results of this study showed that the ratio of T3 to T4 in the exposed group was significantly higher than the control group (*P*= 0.006) (Table 4).

**Table 4:** The ratio of T3 to T4 in study group

***Study Groups***	***N***	***Mean T3/T4***	***Standard Deviation***	***Median T3/T4***	***P-value***
Control	127	.0100	.00356	.0100	0.006
Exposed	371	.0115	.00577	.0108	

## Discussion

The aim of this study was to determine the circulating levels of thyroid hormones 20 years after SM exposure. The mean concentrations of T4 and TSH in the exposed group were not significantly different from the control group. Whereas serum T3 concentrations and T3/T4 ratio exhibited significant increase in SM-exposed people compared to the control group; however, it was within normal clinical range. Severity of injury had no effect on this hormone level. We also assessed TPO, Tg and anti–Tg. The data showed elevated levels of Tg in the SM–not hospitalized exposed group compared to the control group. However, the level of anti–Tg showed no significant differences between the two groups. Anti–TPO was similar in both groups.

To our knowledge, this is the first report pointing to circulating levels of thyroid hormones, 20 years after SM exposure. So far there has been no study on the long-term effects of SM exposure on these parameters to compare.

Serum concentrations of thyroid hormones were measured in the first, third, and fifth week following injury in the SM–exposed group compared to the control group, in order to evaluate the time course of changes in serum concentrations of thyroid hormones, cortisol, and adrenocorticotropic hormone (ACTH) in patients exposed to chemical weapons containing SM (6). In the first week after exposure, free T4 and T3 indices decreased and rT3, cortisol, and ACTH increased. Thereafter, serum hormone concentrations remained unchanged until the fifth week after injury, except for an increase in FT4I, a decrease in TSH by the third week, and a steady decline in serum cortisol. In the current study 20 years following exposure to SM, we found significantly higher levels of T3 in the exposed group compared to the control group, whereas, T4 and TSH levels were not statistically significant. This result is in contrast to acute effects of SM exposure, demonstrated in the foregoing study. The time interval of the post-exposure could be reasonable justification for this dissimilar hormone level. However, other undiscovered pathophysiological mechanisms also should be considered.

Since thyroid function tests in the SM–exposed people in the other studies were assessed short term after exposure, there was no comparable data. Anyway, consistent with the short-term findings (6), no changes were seen in the serum levels of T4 and TSH in our study, 20 years after SM exposure.

The delayed toxic effects of SM were documented in Iranian veterans, focusing on head and neck complications. For the first time, they reported carcinomas of thyroid and nasopharynx in patients with SM exposure (8).

Despite the decrease in T3 level in most of these reports, different elucidations could interpret the elevation of T3 in our study; one is that more subjects in our control group have lower level of T3 than normal range (11.8% in control and 4.3% in exposed). On the other hand, there are numerous human and animal studies on the variety of chemicals that disrupt thyroid hormone homeostasis. Such differences in T3 and T3/T4 ratio were observed in exposure to contaminant chemicals such as PCBs in fish (9).

In vertebrates, the main hormone produced by thyroid, prohormone T4, is converted to T3 in peripheral target tissue cells by 5–deiodinase (10). The differences in T3 and T3/T4 ratio may point to alterations in the peripheral conversion of T4 into T3. Some studies on fish suggested that pesticides and other chemicals may alter 5–deiodinase activity, which can lead to increased T3 and decreased T4 (4–6, 11–13). Although there has been no study on the cellular and molecular mechanisms involved in SM-induced thyroid function disorders, there are a large number of studies on endocrine–disrupting chemicals (EDCs).

Numerous mechanisms have been proposed, including changes in the expression of a large number of potential target genes or proteins, altered by EDCs (14). Activation of EDCs may interfere with thyroid function by interfering with thyroid hormone synthetic pathways, deiodinase functions in peripheral tissues, altering TH turnover, and carrying proteins in the blood (6, 8, 13, 15–19).

However, the limitation of this study is that as our subjects were selected from a previous large cohort study, detailed clinical evaluation in view of specific thyroid assessment was not performed. Further insight into the role of SM in the pathogenesis of thyroid requires deep researches on the cellular and molecular mechanisms involved. This would enable both researchers and clinicians to provide more accurate evaluation and therapeutic strategies when dealing with sufferers of SM exposure.

More studies are needed on the alterations in thyroid hormones, their gene expressions, and involved mechanisms in SM exposure to clarify the causes of these alterations.

## Ethical considerations

Ethical issues (Including plagiarism, informed consent, misconduct, data fabrication and/or falsification, double publication and/or submission, redundancy, etc.) have been completely observed by the authors.
